# Dual anti-cytokine biologic and/or targeted synthetic therapy combination in spondyloarthritis: a narrative review. Are we missing the opportunity for higher remission rates?

**DOI:** 10.3389/fmed.2025.1576411

**Published:** 2025-06-03

**Authors:** Elsa Vieira-Sousa, Pedro Ávila-Ribeiro, João Eurico Fonseca

**Affiliations:** ^1^Faculdade de Medicina, Universidade de Lisboa, Centro Académico de Medicina de Lisboa, Lisboa, Portugal; ^2^Rheumatology Department, Hospital de Santa Maria, Unidade Local de Saúde de Santa Maria, Centro Académico de Medicina de Lisboa, Lisboa, Portugal

**Keywords:** dual combination therapy, biologic DMARD, targeted synthetic DMARDs, bispecific antibodies, dual mechanism of action

## Abstract

Spondyloarthritis (SpA) is a phenotypically heterogeneous group of diseases that share genetic and immune-mediated inflammatory pathways, affecting various organs/and tissues such as the synovium, enthesis, bone marrow, skin, eye, and bowel. Advances in understanding tissue-specific cytokine imbalance in SpA have unveiled an opportunity to foster higher remission rates through a more tailored cytokine blockade. Furthermore, over the years, the accumulated knowledge of the safety profile of approved anti-cytokine treatments has instilled confidence in considering the combination of two cytokine blockade agents for more severe musculoskeletal (MSK) or extra-MSK manifestations/in refractory patients. The rationale for these dual-targeted therapy combination strategies has largely depended on the predominant SpA manifestations and the known efficacy of these therapeutics in monotherapy. More recently, the addition of a targeted synthetic (ts) to a biologic (b) disease-modifying anti-rheumatic drug (DMARD) has also been considered. Additionally, newer bispecific anti-cytokine antibodies and tsDMARDs with dual mechanisms of action have been developed and assessed. Despite limited evidence from randomized controlled trials, real-world data from retrospective cohorts and case series/reports indicate that b/tsDMARD combinations are being used in clinical practice to overcome efficacy limitations of b/tsDMARD monotherapies in more severe either/or difficult-to-treat SpA patients, particularly in the presence of extra-MSK recalcitrant manifestations such as inflammatory bowel disease or psoriasis.

## 1 Introduction

Spondyloarthritis (SpA) is a heterogeneous nosologic entity that encompasses a wide spectrum of axial and peripheral musculoskeletal (MSK) and extra-MSK manifestations affecting the enthesis, joints, bone marrow, skin, bowel, and eye. While sharing some genetic [HLA-B27, interleukin (IL)23 receptor] and pathophysiologic inflammatory pathways [tumor necrosis factor (TNF), IL23, IL17], the individual disease characteristics can be quite diverse, particularly concerning phenotype and severity (1, 2).

While anti-TNF monoclonal antibodies serve as a broad treatment for all manifestations of the SpA spectrum, they are insufficient for many patients to achieve global disease remission. Histopathology and gene expression tissue analyses have shown that, at the tissue level, cytokine relevance can vary across affected organs (3). This is also underscored by the fine-tuned efficacy of available biologics (b) and targeted synthetic (ts) disease-modifying anti-rheumatic agents (DMARDs) that inhibit non-TNF cytokines such as IL17 and IL23.

Despite the available therapies, only 40–50% of patients achieve ASAS40 responses, and <20% a sustained ASDAS remission as targets for treat-to-target in SpA (4), a subset of patients conforms to the most recent definitions of difficult-to-treat SpA/treatment-refractory SpA, for whom standard treatment approaches are clearly insufficient (5, 6).

The combination of conventional synthetic (cs) and bDMARDs is common in clinical practice for certain SpA manifestations, supported by increased efficacy or treatment survival. Furthermore, some combination strategies of csDMARDs have proven to be reasonably safe while providing incremental efficacy (7, 8). The combination of two bDMARDs or a bDMARD and a tsDMARD has been present in the landscape of rheumatology for many years; however, it has been overshadowed by safety concerns. Some unsuccessful combinations for rheumatoid arthritis (RA), such as an IL1 inhibitor (IL1i) combined with a TNF inhibitor (TNFi), have negatively impacted this field in the past (9). Nevertheless, the rationale for combining anti-cytokine biologics (TNFi, IL17i, IL12/23i) and/or tsDMARDs (phosphodiesterase-4 inhibitor (PDE4)/Janus Kinase (JAK) tyrosine kinase (TYK) inhibitors) is attractive in SpA, considering the current understanding of disease physiopathology and the urgent need for better disease control in refractory patients across the wide spectrum of SpA manifestations.

**Figure 1:**
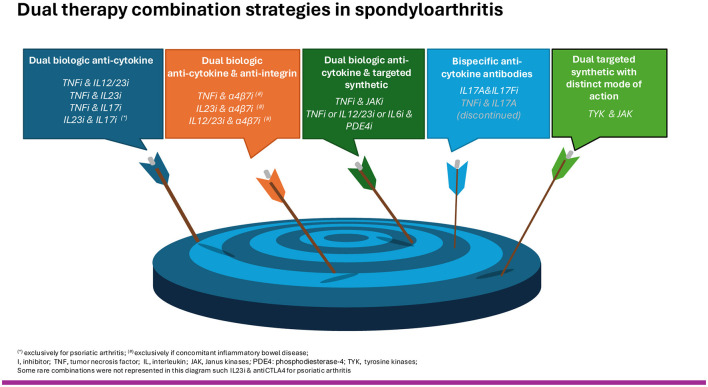
Dual therapy combination strategies in spondyloarthritis.

Herein, we present a narrative review of retrospective clinical cases/series or real-world data and clinical trials on dual-targeted therapies (DTT), defined as a combination of two single-targeted b/tsDMARDs or the use of a newer single b/tsDMARD with a dual mechanism of action, in the field of SpA. We approach SpA as a unifying concept encompassing its wide spectrum of extra-MSK manifestations, including psoriasis (Pso), inflammatory bowel disease (IBD), and uveitis (Figure 1).

We aim to provide the reader with an overview of the most frequent b/tsDMARDs combinations, their indications, efficacy, and safety profile *according* to clinical practice and the most recent evidence on new molecules being assessed with dual-targeted mechanisms in SpA.

## 2 Methodology

A literature review was performed on PubMed up to October 2024, based on the following terms: (“Spondyloarthritis” OR Psoriatic Arthritis” OR “Psoriasis” OR “Inflammatory bowel disease” OR “Ulcerative Colitis” OR “Crohn's Disease” OR “Uveitis”) AND (“dual targeted therapy” OR “combination therapy”) AND (“biologics” OR “biologic therapy” OR “bDMARDs” OR “tsDMARDs” OR “TNF inhibitor” OR “JAK inhibitor” OR “TYK inhibitor” OR “bispecific” OR “adalimumab” OR “apremilast”, “bimekizumab” OR “brepocitinib” OR “brodalumab” OR “certolizumab”, “deucravacitinib” OR “efalizumab” OR “etanercept” OR “golimumab” OR “guselkumab”, “infliximab” OR “secukinumab” OR “sonelokimab” OR “tofacitinib” OR “upadacitinib”, “ustekinumab” OR “remtolumab” OR “risankizumab” OR “vedolizumab”).

We included references from manuscripts written in English from which the full texts were screened. Papers meeting the criteria for DTT—defined as a combination of two anti-cytokine biologic therapies, an anti-cytokine and an anti-integrin biologic therapy, an anti-cytokine and a targeted synthetic drug, as well as new bispecific anti-cytokine antibodies or targeted synthetic drugs with dual mechanisms of action—were selected if they reported on adults (>18 years old) diagnosed with axial spondyloarthritis, psoriatic arthritis, psoriasis, and IBD-associated spondyloarthritis. No specific diagnostic or classification criteria were required in individual studies as inclusion criteria. Study selection and data extraction were independently performed by two of the authors. Efficacy and safety parameters were extracted as they were reported individually in each publication, considering significant heterogeneity to preserve as much information as possible. Broadly used efficacy measures (such as ASDAS for axSpA activity or DAPSA for peripheral PsA activity) were reported in individual columns or in a similar order in the results tables to facilitate comparison. We did not apply restrictions regarding the date of publication or study design: case reports, case series, cohorts, or randomized controlled trials (RCTs). Review publications were used to look for additional references of interest that were not identified in our search strategy. We followed the Scale for the Assessment of Narrative Review Articles (SANRA) principles in the preparation of this manuscript.

## 3 Spondyloarthritis musculoskeletal manifestations (excluding psoriatic arthritis)

### 3.1 Dual biologic anti-cytokine therapy combination

Much of the evidence on dual biologic anti-cytokine therapy combination strategies in SpA MSK manifestations comes from patients with concomitant IBD. In a report of real-world data from 22 SpA patients, 20 with concomitant IBD and a total of 23 drug combinations, the most common was the association of a TNFi and an IL12/23i (17/23) (Table 1) (10). Overall, the main reason for combining biologics was active MSK disease alone (13/23), followed by simultaneous MSK and extra-MSK activity (seven patients, all with IBD) and exclusively active extra-MSK disease (three IBD). The average treatment exposure was 20 months, and all patients had been previously treated with at least one b/tsDMARD.

**Table 1:** Single cohort with individual data on dual biologic anti-cytokine therapy combinations in SpA patients (23 combinations/22 patients).

**References**	**Publication year**	**Disease**	**Indication for DTT**	**Age, sex**	**Previous bDMARD monotherapy**	**Drug 1**	**Drug 2**	**DTT duration (months, median)**	**Efficacy**			**Safety events**	**DTT discontinuation (reason)**
									**ASDAS (6M)**	**ASDAS (last visit)**	**Major improvement**		
Valero-Martinez et al. (10)	2023	SpA	MSK	48y M	IFX, UST	ADA	UST	33	Low	Rem	Yes	No	No
		SpA	MSK	61y F	UST	ADA	UST	17	Low	High	Yes	No	No
		SpA	MSK and IBD	49y M	IFX, CZP, ETA	ADA	UST	16	Rem	Rem	Yes	No	No
		SpA	MSK	41y M	IFX, ADA, CTZ, UST	ADA	UST	13	High	High	No	No	Yes (ineffective IBD and MSK)
		SpA	IBD	56y F	ADA	ADA	UST	14	NA	NA	NA	No	No
		SpA	MSK	47y F	ADA; IFX, UST	ADA	UST	12	Rem	Rem	Yes	No	No
		SpA	MSK	50y M	ADA, IFX	ADA	UST	26	Low	Low	Yes	No	No
		SpA	MSK and IBD	50y M	ADA, IFX, UPA, VDZ, UST	ADA	VDZ^*^	5	NA	High	No	Cytomegalovirus colitis and esophageal candidiasis	Yes (AE)
		SpA	MSK	50y M	ADA, UST	CZP	UST	8	Low	Low	Yes	No	No
		SpA	MSK	55y F	IFX, ADA, ETN, CTZ, UST	CZP	UST	13	Rem	Rem	Yes	No	No
		SpA	MSK	63y M	ADA, IFX, UST	CZP	UST	2	NA	Low	Yes	No	No
		SpA	IBD	41y F	CZP	CZP	UST	15	NA	NA	NA	No	Yes (ineffective IBD)
		SpA	MSK and IBD	40y F	IFX, ADA, CTZ	ETA	UST	41	High	High	Yes	No	No
		SpA	MSK	46y M	ADA, ETN, GOL, CTZ, SEC	ETA	SEC	38	Low	Rem	Yes	No	No
		SpA	MSK	28y M	IFX, ADA, ETN, CTZ, GOL, SEC, TCZ	GOL	SEC	68	Low	Rem	Yes	No	No
		SpA	MSK	32y M	IFX, ADA, ETN, GOL	GOL	RIS	24	Rem	Rem	Yes	No	No
		SpA	MSK and IBD	60y F	IFX, ADA, ETN, SEC, UST	GOL	UST	17	High	Low	Yes	No	No
		SpA	MSK and IBD	40y F	IFX, ADA, CTZ	GOL	UST	1	NA	NA	No	Non-infectious acute hypersensitivity pneumonitis	Yes (AE)
		SpA	MSK	75y M	IFX, ADA, UST	GOL	UST	18	Low	Low	Yes	No	No
		SpA	MSK	22y M	IFX, ADA, ETN, UST, VDZ	GOL	VDZ^*^	20	Rem	Rem	Yes	No	No
		SpA	MSK and IBD	25y M	IFX, ADA, ETN, TOF, TCZ	IFX	TOF^**^	22	Rem	Low	Yes	No	No
		SpA	IBD	38y M	ADA	IFX	UST	16	Low	Low	Yes	No	No
		SpA	MSK and IBD	36y M	IFX	IFX	UST	16	High	High	No	No	No

AE, adverse event, ADA, adalimumab; CTZ, certolizumab pegol; DTT, dual-targeted therapies; ETA, etanercept; GOL, golimumab; IFX, infliximab; NA, not applicable; SEC, secukinumab; TOF, tofacitinib; VDZ, vedolizumab; UST, ustekinumab.

Major clinical improvement (MCI): a change in ASDAS-CRP >2 or improvement greater than 85% in DAPSA.

* Included in this case series although an anti-cytokine and anti-integrin biologic combination.

** Included in this case series although an anti-cytokine biologic and a targeted synthetic drug combination.

The order of DTT therapies considered in the table as drug 1 and drug 2 does not necessarily represent the sequence of introduction.

Regarding efficacy in SpA patients, data were available at 6 months from 21 combinations. At 6 months, 14/21 (66%) of regimens had induced remission or low disease activity according to the axial SpA disease activity score-C reactive protein (ASDAS-CRP < 1.3/ < 2.1, respectively), and throughout the entire follow-up, 17/21 (81%) reached a major clinical improvement (MCI) (change in ASDAS-CRP >2) at some point. Four SpA patients (18%) permanently withdrew from DTT, 2 due to inefficacy (both related to IBD and 1 with concomitant MSK activity) and 2 due to adverse events (AEs).

Only two serious adverse events (SAE) (in two patients) were reported in this population leading to DTT discontinuation: a hypersensitivity pneumonia within the first month of therapy with golimumab (GOL) and ustekinumab (UST) in one patient, and cytomegalovirus colitis and esophageal candidiasis while receiving adalimumab (ADA) and vedolizumab (VDZ) in another patient (10).

### 3.2 Bispecific anti-cytokine biologic therapy

#### 3.2.1 Bispecific anti-IL17A and IL17F antibodies

##### 3.2.1.1 Bimekizumab

The functional role of both IL17A and IL17F may not be redundant in some SpA-affected tissues. The dual inhibition of both IL17A and IL17F has therefore emerged as a potentially effective combination for the treatment of SpA patients (11). Bimekizumab (BKZ) is a monoclonal IgG1 antibody that selectively inhibits both IL17A and IL17F, and its efficacy has been assessed in clinical trials in axial SpA (12), PsA (13), Pso, and hidradenitis suppurativa (14).

In axial SpA (nr-axSpA–MOBILE 1 and r-axSpA–MOBILE 2), 45–48% of patients receiving BKZ 160 mg every 4 weeks achieved ASAS40 at week 16 and 58–61% at week 52, with slightly lower rates in the small subgroup of TNF-inadequate responders. Resolution of peripheral arthritis was observed in 62% and 72%, and of enthesitis in 54% and 51% of nr-axSpA and r-axSpA patients who presented these manifestations at baseline, respectively. The safety profile was consistent with what is known about IL17A inhibitors. During the double-blind therapy period, the most common AEs were nasopharyngitis (9.1/12.1%), upper respiratory tract infection (6.4/9.4%), oral candidiasis (6.1/7.4%), uveitis (2.1/1.2%), and IBD (0.8/0.9%) (12).

Of interest, a network meta-analysis demonstrated a higher relative efficacy of BKZ than secukinumab (SEC) 150 mg and similar efficacy to ixekizumab (IXE) 80 mg in achieving ASAS responses at 12–16 weeks (14). Additionally, an unanchored matching-adjusted indirect comparison of the same therapies at week 52 suggested a higher likelihood of response to BKZ 160 mg than SEC 150 mg, but these differences were non-significant when compared to SEC 300 mg and IXE 80 mg (15).

### 3.3 Dual biologic anti-cytokine and targeted synthetic therapy combination

The combination of a bDMARD and a tsDMARD can enhance effectiveness in SpA by increasing the extent and potency of inflammation reduction through some overlap in the mechanism of action along with distinct cytokine blockade. A retrospective analysis of 15 ankylosing spondylitis patients (29.9±6.72 years) with moderate to high disease activity (ASDAS-CRP 3.82±1.47) despite monotherapy with bDMARDs (4 etanercept (ETA), 3 infliximab (IFX), 5 ADA, 3 SEC) for at least 3 months indicated that adding tofacitinib (TOF) 5mg bid was associated with a significant reduction in ASDAS-CRP (1.47 ± 0.48) at 12 weeks of this combination strategy. The duration of previous bDMARD therapy, however, was not reported. Safety during this short-term period of TOF addition was documented with only two cases of mild gastrointestinal discomfort (one patient on ETA and the other on SEC) and one upper respiratory infection (one patient on ADA) (16). An individual case of the combination of IFX and TOF in a SpA patient with IBD, treated for 22 months and achieving remission at 6 months, was also identified in a pooled description of DTT (10).

## 4 Psoriasis and psoriatic arthritis

### 4.1 Dual biologic anti-cytokine therapy combination

The combination of two cytokine inhibitors for isolated Pso is not commonly found in the literature. Most case reports describe the use of two anti-cytokine bDMARDs in patients with concomitant psoriatic arthritis (PsA). In these clinical cases and case series, the most common regimen reported was the combination of a TNFi and UST or a TNFi and an IL23i or IL17i. Some non-serious adverse events were reported as related to the combination therapy, such as herpes zoster, peritonsillar abscess, and tuberculosis (Table 2).

**Table 2:** Case series/single case reports on dual biologic anti-cytokine therapy combinations in Pso/PsA patients.

**References**	**Publication year**	**Disease**	**Indication for DTT**	**No. of patients**	**Age, sex**	**Previous bDMARD monotherapy**	**Drug 1**	**Drug 2**	**DTT duration (months, median)**	**Efficacy**	**Safety events**	**DTT discontinuation (reason)**
Lowes et al. (81)	2005	Pso/PsA	Pso	1	33y M	ETA, alefacept	EFA	TNFi (IFX)	6	Clinical improvement (not defined)	No	No
Hamilton et al. (82)	2008	Pso/PsA	Pso and PsA	20	Average 50y	ETA (not described to all patients)	EFA	TNFi (ETA/IFX)	16 (8–46)	Various outcomes	5 URI, 1 bronchitis, 1 kidney stone, 1 gastroenteritis, 3 white blood cell elevation, 2 squamous cell carcinoma, 1 basal cell carcinoma, 1 tarsal tunnel syndrome	Not described
Adişen et al. (83)	2008	Pso/PsA	Pso and PsA	1	49y F	EFA	TNFi (ETA)	EFA	1,5	Clinical improvement	No	No
Kitamura et al. (84)	2009	Pso/PsA	Pso and PsA	1	47y F	ETA	EFA	ETA	18	Clinical improvement	Tuberculosis	Not described
Cuchacovich et al. (17)	2012	Pso/PsA	Pso and PsA	1	38y M	IFX, ADA, ETA, ABT, UST,	ABT ETA	ETA UST	Average 7	Significant improvement in PsA composite index (ETA+UST)	No	No
Heinecke et al. (85)	2013	Pso+PsA	Pso and PsA	1	23y F	UST	UST UST	ETA ADA	8/not reported		Furuncles and autoimmune hemolytic anemia	No
Babalola et al. (86)	2015	Pso+PsA	Pso and PsA	1	62y M	IFX, ADA, ETA, UST,	UST T	ETA	6	Both Pso and PsA under control	Unstable angina	No
Gniadecki et al. (87)	2016	Pso+PsA	Pso+PsA	4	Average 50y	IFX, ETA ADA EFA, UST	TNFi (ETA2 or ADA2 or GOL1 or CZP1)	UST	Average 33y	Various outcomes	2/4 had AE: recurrent Herpes zoster, peritonsillar abscess, erysipelas, pneumonia, cellulitis	No (1 and 2 ETA dose reduction to 25mg/w)
Torre and Payette (88)	2017	Pso (palmoplantar)	Pso and PsA	1	33y M	ADA	TNFi (ADA qw)	UST	4	PASI 95	None	No
De Marco et al. (89)	2018	Pso+PsA	PsA	6	Average 47y	ADA IFX, GOL, ETA	TNFi (ETA2, CZP3, ADA1)	UST (6)	Average 12,5	Not reported	Severe skin infection (UST+ETA), URI (UST+CZP/UST+ADA) sarcoidosis Secondary failure and uveitis (UST+CZP) Lack of response	All 6 (4 due to AE: 2 due to lack of efficacy)
Rathod et al. (90)	2019	Pso+PsA	Pso and PsA	1	46y F	ADA,	TNFi (ADA)	GUS	6	PASI 2. No symptoms PsA.	None	No
Thibodeaux et al. (91)	2019	Pso+PsA	Pso and PsA	1	38y F	ADA, IFX, ETA, GOL, UST, SEC, GUS	TNFi (ETA)	+UST +SEC +GUS	15(GUS+ETA)	Minimal disease activity	Increased UTI/URI; hospitalized for H2N1 Flu (UST+ETA)	No
Hanna et al. (92)	2022	Pso+PsA	Pso and PsA	1	48y M	UST, SEC, GOL	TNFi (GOL)	+RIS	12	PASI0, minimal joint pain	None	No

ADA, adalimumab; CZP, certolizumab pegol; EFA, efalizumab; ETA, etanercept; GOL, golimumab; GUS, guselkumab; IFX, infliximab; SEC, secukinumab; TNFi, TNF inhibitor; UST, ustekinumab; RIS, risankizumab; Pso, psoriasis; PsA, psoriatic arthritis; Qw, once a week; UTI, urinary tract infection; URI, upper respiratory infection.

The order considered in the table for DTT as drug 1 and drug 2 does not necessarily represent the sequence of introduction.

Although many of the above reports indicated some improvement in Pso/PsA disease activity with combination regimens, it was not possible to infer a preferable association based on either efficacy or safety patterns. As expected, in some particularly severe cases, secondary failures were also observed. Other combinations, such as those of abatacept (ABT) and ETA, were reported as unsuccessful (17).

From the same real-world study previously described in the SpA subchapter, a subgroup of 14 PsA patients (5 with concomitant IBD) identified a total of 16 drug combinations (Table 3). The reasons for combining biologics were peripheral and/or axial MSK activity (7/16), both MSK and extra-MSK activity (7/16)—of which six were due to active IBD and one to active uveitis—and exclusively active extra-MSK disease activity (one IBD and one PsO). The average duration on DTT was 10.5 months. The combination of TNFi with an IL17i (6/16), an IL12/23i (4/16), or an IL23i (1/16) were the most frequently reported. In comparison to SpA, PsA patients had been exposed to a higher number of b/tsDMARDs (5±3 vs. 3±2). At 6 months, 4/15 (27%) of DTT regimens led to remission/low disease activity, as assessed by the disease activity in psoriatic arthritis score (DAPSA <4/<14, respectively). In addition, at least at one time point in follow-up, 8/15 (53%) reached an MCI (improvement >85% in DAPSA). There was one SAE in this population, a staphylococcal bacteremia at 8 months of DTT in a PsA patient with cirrhosis and multiple comorbidities. One patient interrupted DTT due to pregnancy (10).

**Table 3:** Single cohort with individual data of dual anti-cytokine biologic combination therapy in PsA patients (16 combinations/14 patients).

**References**	**Publication year**	**Disease**	**Indication**	**Age, sex**	**Previous bDMARD monotherapy**	**Drug 1**	**Drug 2**	**DTT duration (months, median)**	**Efficacy**			**Safety events**	**DTT discontinuation (reason)**
									**DAPSA/ASDAS (at 6M)**	**DAPSA/ASDAS(at last visit)**	**Major improvement**		
Valero-Martinez et al. (10)	2023	PsA	Axial MSK + IBD	70y M	IFX	ADA	UST	12	High	High	No	No	Ineffective MSK and IBD
		PsA	Peripheral and axial MSK + IBD	32y M	IFX, UST	ADA	UST	2	NA	NA	No	Pregnancy	No
		PsA	Peripheral and axial MSK + IBD	32y M	IFX, UST	CZP	UST	5	High	NA	No	No	Ineffective MSK
		PsA	Peripheral and axial MSK + IBD	37y F	ADA, IFX, UST	CZP	UST	19	Low	Low	Yes	No	No
		PsA	Peripheral MSK	44y F	ADA, ETN, IFX, SEC, IXE, UST, GUS	CZP	GUS	9	Low	Low	Yes	No	No
		PsA	Peripheral MSK	64y M	IFX, ADA, ETN, GOL, SEC, IXE	ADA	SEC	6	Moderate	High	No	No	Ineffective MSK
		PsA	Peripheral MSK	66y F	IFX, ADA, IXE	ADA	IXE	9	Moderate	Moderate	Yes	No	Ineffective MSK
		PsA	Peripheral and axial MSK	62y F	IFX, ETN, GOL, SEC	ETA	SEC	8	Moderate	Low	Yes	Staphylococcal bacteriemia	No
		PsA	Psoriasis	34y F	IFX, ADA, ETN, CTZ, GOL, SEC, UST	ETA	SEC	12	NA	NA	NA	No	No
		PsA	Peripheral and axial MSK	61y F	IFX, ADA, ETN, CTZ, SEC, IXE, TOF	GOL	SEC	26	Low	Low	Yes	No	No
		PsA	Peripheral MSK	40y F	ADA, ETN, IFX, SEC, IXE, UST, GUS,	GOL	BRO	8	Row	Low	Yes	No	No
		PsA	Peripheral and axial MSK and uveitis	49y F	ADA, IFX, CTZ, GOL, IXE, UPA	GUS	IXE	9	Moderate	Moderate	Yes	No	No
		PsA	Peripheral and axial MSK	54y M	ADA, IFX, SEC, UST, GUS	GUS	ABA^*^	21	Moderate	Low	Yes	No	Patient decision
		PsA	Axial MSK + IBD	70y M	IFX	ADA	VDZ^**^	3	High	High	No	No	Ineffective MSK and IBD
		PsA	Peripheral and axial MSK + IBD	58y F	ETN, ADA, CTZ, UST, SEC, VDZ	GUS	VDZ^**^	5	NA	High	No	No	Ineffective IBD
		PsA	IBD	58y F	ETN, ADA, CTZ, UST, SEC, VDZ	UST	VDZ^**^	14	High	High	No	No	Ineffective MSK and IBD

ABT, abatacept; ADA, adalimumab; BROD, brodalumab; CZP, certolizumab pegol; ETA, etanercept; GOL, golimumab; GUS, guselkumab; IFX, infliximab; IXE, ixekizumab; NA, not applicable; SEC, secukinumab; VDZ, vedolizumab; UST, ustekinumab.

Major clinical improvement (MCI): a change in ASDAS-CRP >2 or improvement greater than 85% in DAPSA.

* Included in this case series although an anti-cytokine and an anti-CTLA4 biologic combination.

** Included in this case series although an anti-cytokine and an anti-integrin biologic combination.

The order considered in the table for DTT as drug 1 and drug 2, does not necessarily represent the sequence of introduction.

Despite this encouraging evidence, due to its limited quality, results from a phase 2 RCT designed to assess the efficacy of guselkumab (GUS) in combination with GOL compared to GUS monotherapy (AFFINITY) in PsA are awaited. This trial defined the primary endpoint as the number of PsA patients who achieve minimal disease activity (MDA) score at 24 weeks.^1^

Although outside the scope of this article, it is important to note that sequential IL23i and IL17i therapy (GUS 100 followed by SEC 150 mg after 2 months, followed by GUS 100 mg after 1 month) has also been reported, based on the rationale that IL23 might have long-lasting effects and sensitize patients for IL17i therapy. This has been described, for example, in three PsA patients refractory to ADA, SEC, and GUS. All three patients achieved MDA at 6 months with no adverse events reported (18).

### 4.2 Bispecific anti-cytokine biologic therapy

#### 4.2.1 Bispecific anti-TNF and IL17 antibodies

##### 4.2.1.1 Remtolumab

ABT-122, a bispecific monoclonal antibody that neutralizes both TNF and IL17A, was the first of this class to be developed, initially assessed in PsA in a phase 2 trial (240 PsA patients) and in RA (16, 19, 20). The results from the PsA trial showed overall similar American College of Rheumatology (ACR) 20/50/70 and PASI responses between ABT-122 (120 or 240 mg) every week and ADA 40 mg every other week, at 12 weeks of treatment in bio-naïve PsA patients. Superiority to ADA was observed with the 240 mg dose for ACR50/70 and PASI 75. The safety profile of ABT-122 was acceptable, and in fact, AEs were similar to those of ADA during the placebo-controlled period, and this was generally maintained for up to 36 weeks in the open-label extension (20). Nevertheless, even though superiority for bispecific TNF&IL17 blockade vs. TNF monotherapy was not confirmed, this trial showed that dual TNF and IL17 inhibition was not associated with an increased risk of AEs. Despite these favorable results, the development of ABT-122 in PsA was halted.

#### 4.2.2 Bispecific anti IL17A and IL17F antibodies

##### 4.2.2.1 Bimekizumab

BKZ clinical trials program in Pso included the initial phase 2 RCTs BE ABLE1 and 2, the phase 3 dose escalation BE READY (21), and the comparative head-to-head trials BE RADIANT (with SEC) (22), BE SURE (with ADA) (23), and BE VIVID (with UST) (24). In these head-to-head trials, BKZ showed clear separation in efficacy from biologics with other mechanisms of action, achieving higher rates of the Psoriasis Area Severity Index (PASI) 100 than SEC (75% vs. 53% at w48), ADA (62% vs. 26% at w16), and UST (65% vs. 38% at w52). This was further confirmed in a systematic literature review and network meta-analyses of RCTs on the efficacy of BKZ in the short term (10–16 w) in patients with moderate to severe Pso treated with BKZ compared to other bDMARDs. This analysis demonstrated that BKZ 320 mg had the highest probability of inducing PASI 90 and 100 compared to TNFi, IL23i, and IL17i. Real-world data also corroborates the high effectiveness of BKZ in distinct populations, with a similar safety profile to that observed in RCTs (25, 26). Notably, there were no differences in response rates between bio-naive and bio-experienced patients in these studies. In a broader spectrum of SpA-associated skin manifestations, the phase 3 trials BE HEARD I and II also showed that BKZ was able to induce a clinical response of at least 50% in 48% and 52% of patients with moderate-to-severe hidradenitis suppurativa (27).

In PsA patients, phase 2b escalating dose (28) studies, including BE OPTIMAL in bDMARDs-naïve patients with an active reference arm (ADA) (29), and BE COMPLETE (along with its long-term extension, BE VITAL) in trials with inadequate response or intolerance to TNFi, showed sustained high efficacy through follow-up (13). In BE OPTIMAL, ACR50 was 44% for BKZ 160 mg vs. 46% for ADA at week 16, and 54% for BKZ 160 mg vs. 50% for ADA at week 52, observed in bDMARDs-naïve patients. In BE COMPLETE, ACR50 was 43% for BKZ 160 mg at week 16 and 52% at week 52 in the TNFi IR population. The most frequent treatment-emergent adverse events (TEAEs) in BE COMPLETE were SARS-CoV-2 (COVID-19), oral candidiasis, nasopharyngitis, and urinary tract infection. In BE OPTIMAL, the rate of TEAEs was similar between BKZ and ADA, but there was an increased frequency of Candida infections (7.7%) during BKZ treatment compared to 1 (0.7%) during ADA treatment. An attempt was made to indirectly compare the bicytokine IL17A/F blockade with a monocytokine IL23 blockade using a matching-adjusted indirect comparison. Results from the comparison of BE OPTIMAL and DISCOVERY 2 for the bDMARD-naïve population, as well as from BE COMPLETE and COSMOS for the TNFi-IR population, demonstrated that BKZ 160 mg Q4W was associated with a higher likelihood of achieving ACR50 at week 52 than GUS 100 mg Q4W in bDMARDs-naïve patients (30). Furthermore, in a network meta-analysis comparing BKZ with approved b/tsDMARDs (16 treatments), pairwise comparisons favored BKZ in TNFi-IR for ACR20 and ACR70 responses, ranking first in this subgroup of patients. In b/tsDMARDs-naïve patients, the effect favored ETA, IFX, and GOL for ACR responses, but for more stringent criteria such as MDA, BKZ again ranked first (31).

##### 4.2.2.2 Sonelokimab

Sonelokimab (SNL) is a trivalent nanobody bispecific IL17A and IL17F inhibitor. As a nanoparticle, better tissue penetration is expected. SNL was initially studied in Pso in a dose escalation phase 1 trial. The results from this trial showed PASI 90 and 100 responses in 100% and 56% of patients, respectively, at day 85 for those treated with SNL 240 mg every other week. The effects were noted as early as the first week of treatment (32). In the phase 2b trial, an investigator global assessment scale score of 0 or 1 was achieved by 77.4% of the SNL 120 mg normal load group (induction SNL at weeks 0, 2, 4, and 8), 88.2% of the SNL 120 mg augmented load group (induction SNL at weeks 0, 2, 4, 8, and 10), and 77.4% of the SEC 300 mg comparison group. Regarding safety, AEs were observed in 49.5% of patients, with a slightly higher incidence in the SNL group during the first 12 weeks of treatment. The most common AEs were nasopharyngitis (13.5%), pruritus (6.7%), and upper respiratory tract infections (4.3%). Notably, during the remaining trial period (12–52 weeks), mycotic infections caused by *Candida* were more frequent in the SNL group (7.4%) vs. to the SEC group (1.9%) (33). A phase 2 clinical trial assessing SNL in active PsA has concluded, but results are still awaited, and phase 3 trials are expected. Similarly, in moderate to severe hidradenitis suppurativa, phase 2 trials have concluded, and phase 3 trials are ongoing, with results yet to be published.^2^

### 4.3 Dual anti-cytokine biologic and targeted synthetic therapy combination

The evidence on the association of the phosphodiesterase-4 inhibitor (PDE4) apremilast and bDMARDs for Pso, although scarce and based on retrospective studies (total of 172 patients), appears to show a good safety profile, with mostly mild gastrointestinal adverse events, similar to monotherapy, as recently described in a systematic literature review (34). Some efficacy improvements with this combination, in a small cohort of patients with bDMARDs secondary failures, have also been noted at 24 months in Pso patients (35). Based on retrospective data, 71 DMARD-refractory PsA patients treated with apremilast 30 mg bid monotherapy (39 patients) or combination therapy (32 patients) either with csDMARDs (24 patients) or with bDMARDs (8 patients) (CZP, GOL2, ADA, ETA, UST, SEC, or tocilizumab-TCZ) experienced no increase in the number of AEs between the two groups. Among those 51 patients with >6 months of follow-up, a slightly higher proportion of patients in the apremilast monotherapy group (65 vs. 57 in the combination group) achieved a clinical response, but the exposure to previous bDMARDs was proportionally higher in the combination group (71% vs. 51% in the monotherapy group), likely reflecting more severe patients (36). Another retrospective study of 22 patients with Pso and PsA reports the safety of combining apremilast with ongoing bDMARDs, with six patients experiencing minor adverse events [nausea (2), diarrhea (2), weight loss (1), and abdominal pain (1)]. Improvements in skin condition and pain were observed in more than 50% of patients, as well as reductions in CRP levels (37).

Some case series on the combination of TOF and bDMARDs in refractory PsA patients have also been published. The earliest describes a female patient previously refractory to 4 bDMARDs and 1 tsDMARD who was treated with a combination of TOF and TCZ. At 28 months, this treatment was discontinued due to lack of efficacy (38). In the second report, different combinations of TOF with RIZ (1 patient), GUS (1 patient), UST (1 patient), IXE (2 patients), and SEC (1 patient) were described. In all cases, the addition of TOF to the ongoing bDMARD resulted in an improvement in efficacy, although arthritis remission was not achieved in all (39).

The safety and efficacy of a combination of the TYK2 inhibitor deucravacitinib with standard bDMARDs for Pso and PsA have also been described. A total of 20 patients, previously treated with an IL17i or IL23i for more than 6 months, were included: 12 due to worsening of PsA and the remaining due to Pso activity (BSA > 5%). Among those included due to PsA activity, 5/out of 12 experienced an improvement in the Psoriatic Arthritis Impact of Disease (PSAID) >1, while the other half reported an improvement of <1, and two felt worsening of their PsA despite treatment. In addition, most patients included due to refractory Pso experienced a beneficial effect on skin lesions. Three of 20 patients discontinued treatment during the 3 months of this study, two due to gastrointestinal symptoms, and one due to difficulties in accessing medication. Another patient reported worsening of acne that did not lead to treatment suspension. The safety profile of this combination regimen was shown to be reasonable and appears to provide potential incremental therapeutic effects for both PsA and Pso patients (40).

### 4.4 Targeted synthetic therapy with a dual mechanism of action

Brepocitinib is a dual selective inhibitor of TYK2 and JAK1, expected to provide greater efficacy than oral therapies targeting either TYK2 or JAK1 alone. It is being studied in several immune-mediated diseases, including PsA. The results from the phase 2b randomized placebo-controlled trial in active PsA showed ACR20 response rates of 66.7% and 77.4% for the 30 mg and 60 mg once-a-day doses, respectively, vs. to 43.3% of patients in the placebo arm. SAEs were observed in 5.5% of patients and included infections in 6 participants (2.8%). No comparative studies with other ts or bDMARDs are yet available (41).

## 5 Inflammatory bowel disease

Inflammatory bowel disease (IBD), alone or in association with SpA, is the setting where DTT has been used more frequently for several reasons. First, bDMARD and tsDMARD options for the treatment of IBD have historically been scarcer than for SpA and PsA, making DTT a more appealing alternative to using higher dosages of single drugs in cases of refractory disease. Second, the potentially disabling or life-threatening short-term complications of poorly controlled IBD make “aggressive” treatment often more urgent than in SpA or Pso/PsA. Lastly, distinct manifestations of the SpA/IBD disease spectrum may respond differently to drugs, prompting the use of DTT to treat concomitant disease manifestations. For instance, as is well known, anti-integrin antibodies natalizumab (NAT) (anti α4β1) and VDZ (anti-α4β7) have no efficacy on extraintestinal manifestations (EIM), and IL17i are effective for the treatment of axial and peripheral SpA but not for IBD. Indeed, the simultaneous occurrence of active IBD and EIM has been reported as one of the main reasons for the use of DTT, along with refractory disease.

### 5.1 Dual biologic anti-cytokine and/or anti-integrin therapy combination

Three trials (2 RCTs and one single-arm prospective trial) assessed DTT for the treatment of refractory IBD, combining a TNFi with an anti-integrin antibody or IL23i. The first report focused on the association of IFX and NAT in Crohn's disease (CD) (42). A non-significant decrease in CD Activity Index (CDAI) score was observed in patients taking NAT in addition to IFX compared to those taking IFX alone, with a similar safety profile. However, concerns over a small but potentially lethal risk of progressive multifocal leukoencephalopathy associated with NAT led the U.S. Food and Drug Administration to issue a boxed warning for this drug, and it was never approved for use in IBD. More recently, the VEGA trial compared the combination therapy of GUS plus GOL against GUS or GOL monotherapy in patients with ulcerative colitis (UC) (43). Including 214 participants across the three treatment arms, this was the largest trial of DTT in IBD. Eighty-three percent of patients in the GUS plus GOL group achieved clinical response (≥30% decrease from baseline in the full Mayo score and a decrease of ≥3 points with either a decrease in rectal bleeding score of ≥1 point or a rectal bleeding score of 0 or 1) at week 12, compared with 61% in the GOL monotherapy group (*p* =0.0032) and 75% in the GUS monotherapy group (*p* = 0.2155). AEs were frequent (63–76% across treatment arms at week 50) but overall similar across groups and less frequent with DTT (63%). Infections occurred in 31%, 32%, and 24% of patients treated with DTT, GOL, and GUS monotherapy, respectively; serious infections were similarly observed in 6% of patients in each group. Lastly, the EXPLORER trial studied the combination of ADA and VDZ in 55 CD patients. This was a single-arm (ADA + VDZ + methotrexate), open-label study that included a meta-analysis of previous placebo and biologic monotherapy trials, along with a *post hoc* Bayesian analysis to compare the observed endoscopic remission rates with those previously reported for monotherapy (104). DTT achieved endoscopic remission in 33.5% of participants, and the probability of being superior to VDZ monotherapy (27% endoscopic remission rate) or ADA monotherapy (30% endoscopic remission rate) was 86.3% and 71.4%, respectively. A total of 87.3% of patients experienced AEs, mostly arthralgia and worsening of CD (16.4% each); six patients (10.9%) had SAEs, including infections and complications of CD.

Regarding SpA MSK manifestations, only the VEGA trial reported the presence of arthralgia and arthritis in 13.6% and 5.1% of participants at baseline, respectively, while the EXPLORER trial described arthralgia as a side effect of VDZ treatment. However, the efficacy of DTT on EIM was not mentioned in any of the aforementioned trials.

Despite the lack of data about SpA manifestations, large trials of DTT in IBD provide valuable insights into the safety of DTT, especially for the combination of TNFi and IL23i, which is also used in the context of SpA or PsA in the absence of IBD, as previously described.

To gain insight into the use of DTT for SpA associated with IBD and the efficacy of DTT on SpA manifestations, one must rely on observational evidence (Table 4). Most studies report one or two cases of successful use of DTT for either refractory IBD or EIM, or less frequently, both refractory IBD and EIM occurring simultaneously. AEs were seldom reported and mostly mild. This contrasts with the two largest case series of bDMARD DTT focused on refractory IBD, where only around 30% achieved endoscopic remission and up to 50% were considered clinical responders, highlighting the publication bias for single positive case reports (44, 45). Case series of DTT for the treatment of EIM were more reassuring, reporting significant improvement of EIM (including SpA) in over 75% of patients. One notable exception was paradoxical PsO associated with TNFi, which did not improve with the addition of UST unless the offending TNFi was stopped (46). More AEs were also described in case series (up to 30% of patients), mostly infections, compared to only one case report disclosing a self-limited viral infection (47). The authors consistently stated that these AEs were not observed more frequently than with single bDMARD therapy.

**Table 4:** Case series/single case reports on the combination of dual biologic anti-cytokine and/or anti-integrin and/or targeted synthetic therapies in IBD/SpA patients.

**First author**	**Publication year**	**Disease**	**Concomitant SpA**	**Indication for DTT**	**No. of patients**	**Drug 1**	**Drug 2**	**DTT duration (months, median)**	**Efficacy**	**Safety events**	**Discontinuation of DTT (reason)**
**bDMARD combination only**											
Hirten et al. (93)	2015	CD	No	EIM only	1	IFX	VDZ	2	Clinical and luminal disease improvement, resolution of erythema nodosum	No	1 (VDZ withdrawn due to IBD remission).
Yzet et al. (46)	2016	CD+ UC	No	EIM only	3	TNFi	UST	21	IBD in remission under TNFi monotherapy, 3/3 patients who developed paradoxical psoriasis were treated with TNFi+UST and had no cutaneous improvement until TNFi withdrawn.	No	3 (TNFi and UST withdrawn due to paradoxical Pso not responsive to UST)
Bethge et al. (51)	2017	UC	Yes	EIM only	1	ETN	VDZ	10	Clinical, endoscopic and histological remission of UC, clinical remission of axSpA	No	No
Liu et al. (94)	2017	CD	No	RD	1	UST	VDZ	6	Clinical and endoscopic remission	No	No
Huff-Hardy et al. (47)	2017	CD	No	RD	1	UST	VDZ	12	Clinical and endoscopic remission	1 rotavirus infection	No
Fischer et al. (95)	2017	UC	Yes	RD+EIM	1	CTZ	VDZ	21	Clinical, endoscopic and histological remission of UC, clinical remission of SpA	No	No
Buer et al. (96)	2018	CD+ UC	Yes	RD	10	TNFi	VDZ	17	10/10 patients achieved clinical remission.	2 URI	8 (7 withdrew TNFi and 1 both TNFi and VDZ due to IBD remission)
Mao et al. (97)	2018	CD	Yes	RD, EIM	4	TNFi/UST	VDZ	15	3/4 patients achieved clinical remission of CD. 1 patient with SpA (ETN+VDZ and later ETN+UST) achieved SpA remission, but CD remained active	2 *Clostridium difficile* infections (same patient), 2 hand–foot–mouth and influenza infections	No
Roblin et al. (98)	2018	UC	Yes	EIM only	1	GOL	VDZ	12	Clinical and endoscopic remission of UC, clinical remission of SpA	No	No
Richard et al. (52)	2018	CD+ UC	Yes	RD	2	CTZ	VDZ	3,5/10	Patient 1: Clinical remission of CD and SpA. Patient 2: inaugural flare of SpA after initiation of VDZ, SpA remission but flare of UC after switch to CTZ, SpA recurrence with the combination of VDZ and CTZ	No	1 (CTZ withdrawn due to SpA activity).
Cline et al. (99)	2019	CD	No	EIM only	1	ADA	UST	>1	“Clear improvement” of hidradenitis suppurativa and CD	No	No
Yang et al. (44)	2020	CD	No	RD	22 patients/24 trials of DTT	Any combination of TNFi/UST/VDZ		9	Endoscopic improvement (>50% reduction in SES-CD) occurred in 43% of trials and 26% achieved endoscopic remission. 50% had clinical response and 41% achieved clinical remission. Among the combinations used VDZ+UST had numerically higher rates of endoscopic improvement but with similar endoscopic remission and adverse event rates.	AE in three trials (13%). 1 trial ended due to drug-induced lupus attributed to adalimumab. 1 patient pneumonia and another basal cell skin cancer, recurrent *Clostridium difficile* infection and *Acinetobacter bacteremia* (recurrent history of all three diseases prior to initiation of DTT)	15 (>50% due to IBD activity).
Privitera et al. (100)	2020	CD+ UC	Yes	RD, EIM	16	Any combination of TNFi/UST/VDZ; 1 VDZ+apremilast and 1 VDZ+secukinumab		7	Clinical response (of intestinal or extra-intestinal symptoms, according to the indication for DTT) in 100% of patients by the end of the induction. Four patients discontinued DTT during follow-up (2 treatment failure, 1 clinical remission, 1 loss to follow-up).	AE in 18.8% of patients, all non-serious	4 (2 to IBD/SpA activity, 1 due to remission, and 1 cutaneous reaction)
Biscaglia (101)	2020	CD+ UC	No	RD	2	UST	VDZ	21 and 24	2/2 patients achieved clinical remission of IBD and Pso.	No	2 (1 VDZ and 1 UST withdrawn due to remission)
Elmoursi et al. (54)	2020	CD	No	RD	1	UST	VDZ	>1	Clinical and endoscopic remission.	No	No
Fumery et al. (102)	2020	CD+ UC	1	RD+EIM, EIM only.	7	TNFi/Ocrelizumab	UST/VDZ	6	4/7 patients achieved deep remission and 2/7 patients achieved clinical and biological remission of IBD. Patients with SpA: patient 1: UST+GOL (IBD: clinical and biological remission, endoscopic response; AS: clinical remission); patient 2: ETN+VDZ (IBD: deep remission; AS: clinical response). 1/1 patient with multiple sclerosis obtained clinical remission with ocrelizumab (and IBD clinical and biological remission with VDZ).	No	4 (1 withdrew TNFi and UST due to IBD activity, 3 withdrew UST due to non-responsive paradoxical Pso [same as (46)]
Kwapisz et al. (103)	2021	CD+ UC	No	RD	15	Any combination of TNFi/UST/VDZ		24	11/15 patients (73%) reported symptomatic improvement, 10 patients (67%) had reduction of corticosteroid use, and 4 patients (44%) had endoscopic or radiographic improvement.	4 patients (27%) had infections requiring antibiotics, 3 patients were hospitalized, and 3 patients (20%) required surgical intervention 1 patient discontinued VDZ because of arthralgias	1 (VDZ withdrawn due to arthralgias)
Eronen et al. (45)	2022	CD+ UC	1	RD	16 patients/22 trials of DTT	Any combination of TNFi/UST/VDZ. Mostly TNFi+UST (n=10; 45.5%).		9	7 patients (32% of trials) achieved clinical and endoscopic remission, 2 trials (9%) achieved partial response. 4/10 trials reduced the need for corticosteroids.	3 patients (19%) had infections requiring antibiotics	9 DTT trials stopped (IBD activity)
**bDMARD and tsDMARD combination**											
Le Berre et al. (60)	2019	UC	1	RD+EIM	1	TOF	VDZ	3	Clinical remission of UC and SpA.	No	No
Glassner et al. (57)	2020	CD+ UC	1	RD, EIM	50 patients/53 trials of DTT	Any combination of TNFi/TOF/UST/VDZ, mostly UST+VDZ (n=25, 47.2%); 20 combinations included TOF (37.7%) and 1 apremilast (1.9%)		8	Significantly more patients in clinical and endoscopic remission at follow-up than at baseline (50% vs 14%, P = 0.0018; and 34% vs 6%, P = 0.0039, respectively). 66% (19/29) were able to discontinue steroids. 3 patients started DTT for EIM, but effectiveness on EIM was not reported.	13 patients (26%) experienced adverse events, 78% of which occurred on concomitant steroid treatment. 6 patients (12%) had serious adverse events, all of the non-lethal infections	Not detailed.
Goessens et al. (58)	2021	CD+ UC	1	RD, EIM, RD+EIM	98 patients/104 trials of DTT	Any combination of TNFi/TOF/VDZ/inhibitors of IL4/13, IL5, IL6, IL12/23, IL17A, and IL23 16 combinations with other molecules: apremilast, ciclosporine, rituximab, leflunomide, and tacrolimus.		8	Clinical improvement of IBD in 70% and EIM/concomitant disease activity in 81% of the patients (DTT started for RD in 67%, for EIM or concomitant disease in 22%, for both in 10%). Overall, DTT was continued in 55% of the patients.	42 significant adverse events were observed (42% of patients), mostly related to uncontrolled IBD. 10 severe but non-lethal infections (60% on concomitant steroids and/or immunomodulators, 90% with TNFi)	47 (25 due to IBD activity, 5 due to EIM activity, 10 due to improvement, 2 due to intolerance, 4 due to adverse events, and 1 due to patient's decision)
Miyatani et al. (59)	2024	CD	1	RD, EIM, RD+EIM	10	UPA	UST	10	RD: 7/8 patients achieved clinical remission. EIM: 3/4 improved joint pain.	7/10 patients experienced adverse events, mostly mild upper respiratory infections and nausea. 1 patient had to stop DTT due to nausea and cutaneous fungal infection.	1 (due to nausea and cutaneous fungal infection)

ADA, adalimumab; AS, ankylosing spondylitis; axSpA, axial spondyloarthritis; bDMARDs, biologic disease-modifying agents; CD, Crohn's disease; CDAI, Crohn's Disease Activity Index; CRP, C-reactive protein; CTZ, certolizumab; DTT, dual-targeted therapy; EIM, extraintestinal manifestations; ETN, etanercept; FU, follow-up; GOL, golimumab; HBI, Harvey–Bradshaw Index; IBD, inflammatory bowel disease; IFX, infliximab; PsA, psoriatic arthritis; Pso, psoriasis; RA, rheumatoid arthritis; RD, refractory disease; SES-CD, Simple Endoscopic Activity Score in Crohn's Disease; SpA, spondyloarthritis; TNFi, TNF inhibitor; TOF, tofacitinib; tsDMARDs, targeted synthetic disease-modifying agents; UC, ulcerative colitis; UPA, upadacitinib; UST, ustekinumab; VDZ, vedolizumab.

The order considered in the table for DTT as drug 1 and drug 2, does not necessarily represent the sequence of introduction.

Both IBD flares after starting treatment of SpA with ETA (48) or SEC (49) and SpA flares after starting treatment with VDZ for IBD (50) have been described, even though it remains unclear if there is a causal relationship or if these are simply new manifestations of the disease unrelated to treatment. Interestingly, DTT with ETA was able to control worsening SpA manifestations after VDZ initiation while maintaining remission of previously controlled IBD (51). Similarly, adding VDZ to CTZ pegol for uncontrolled IBD did not worsen previously controlled SpA. In the event of SpA being precipitated by VDZ, however, switching to a TNFi was able to control SpA, but not if both TNFi and VDZ were combined (52).

It is worth noting that many case reports refer to severely refractory diseases, often with four or more prior biologic treatment exposures. Despite this, most case reports described successful control of IBD and/or EIM while keeping the previously ineffective treatment, combined with a new drug or even combining it with another drug that had also been previously ineffective when used alone (47, 53, 54). In line with this finding, it is likely that the contrast between the modest benefit of DTT observed in clinical trials and the overwhelming remission rates described in observational studies and case reports is related to differences in the study population, namely disease refractoriness and EIM. Clinical trials included mostly patients with no EIM (or in whom EIM were not a reason for DTT) and biologic-naïve patients (EXPLORER) or those with little exposure to b/tsDMARDs (NAT trial patients had up to 3 months of exposure to IFX; VEGA participants were allowed to have previous exposure to VDZ or TOF only). Observational data suggest that patients with IBD refractory to multiple b/tsDMARD lines or with simultaneous intestinal manifestations and EIM not controlled with a single agent will benefit the most from DTT.

### 5.2 Bispecific anti-cytokine biologic therapy

The potential of bispecific antibodies for the treatment of IBD has been recognized and recently discussed by leading experts in an editorial (55). A bispecific antibody targeting IL1β and IL17A in an IBD model in mice has shown promising results (56). To our knowledge, however, they have not been tested on humans so far.

### 5.3 Dual biologic and targeted synthetic therapy combination

Only four publications included patients treated with tsDMARDs as part of DTT (Table 4), but these comprise the two largest cohorts of DTT described to date. One of these reviews includes 20 DTT trials featuring TOF, mostly in combination with TNFi (n = 9) and VDZ (*n* = 8) (57), while the other reports 13 trials of TOF + VDZ and 1 trial of TOF + TNFi (58). The first does not discriminate efficacy results by class of DTT, but the second states that the TOF + anti-integrin combination had the second highest chance of success in UC (8/12; 67%) after the TNFi + anti-integrin combination (8/11; 73%). In this same second review, three patients started DTT with TOF for EIM, but the efficacy for these patients was not detailed. None of the 18 serious AEs reported in both series occurred in patients taking TOF.

One study accounted for 10 patients treated with upadacitinib (UPA), all in combination with UST. Seven of eight (88%) of those with refractory CD achieved clinical remission, while three out of four (75%) patients treated with DTT for arthralgia (1 PsA patient, the other three with no previous SpA diagnosis) improved joint pain (59). Seventy percent of patients experienced AEs (by far the highest rate reported, possibly due to more strict reporting criteria), but only one had to stop DTT because of nausea and a cutaneous fungal infection (the non-responding arthralgia patient).

Apart from the three series mentioned above, three more single case reports achieved clinical remission of previously active axial or peripheral SpA with a combination of TOF and VDZ (60, 61).

### 5.4 Targeted synthetic therapy with dual mechanism of action

Brepocitinib (an oral TYK2/JAK1 inhibitor) has been assessed in comparison to ritlecitinib (an oral JAK3/TEC family kinase inhibitor, already approved for the treatment of severe alopecia areata) as induction therapy for active, moderate-to-severe UC (62). In this phase 2b randomized placebo-controlled trial (VIBRATO), the placebo-adjusted proportions of patients with modified clinical remission at week 8 were 13.7%, 32.7%, and 36.0% for ritlecitinib 20, 70, and 200 mg, respectively, and 14.6%, 25.5%, and 25.5% for brepocitinib 10, 30, and 60 mg, respectively. Infections were observed in 16.9% of patients treated with brepocitinib, compared to 8.7% with ritlecitinib and 4.0% with placebo. Serious AEs occurred in 3.5% of patients treated with brepocitinib and 4.0% with ritlecitinib (compared to 0% with placebo). Results from a similar trial in CD (PIZZICATO) have recently been presented and are expected to be published soon. Results from the longer-term extensions of these studies will be needed, as well as studies with larger sample sizes, to clarify the safety profile of these drugs (63).

## 6 Uveitis

Studies on the therapeutic effect of b/tsDMARDs in SpA-associated acute anterior uveitis (AAU) are scarce. There is currently no formally approved therapy for SpA-associated AAU. Nevertheless, ADA—indicated for intermediate, posterior, or panuveitis—is commonly used off-label for AAU. Most of the available evidence for this extra-MSK manifestation comes from observational retrospective data and secondary endpoints from SpA RCTs, with only one multicenter open-label trial having uveitis as the primary endpoint (64, 65). The ability to reduce the recurrence of AAU flares appears to be higher for TNFi monoclonal antibodies (particularly IFX and ADA) than for IL17i, based on retrospective analyses and a pairwise and network meta-analysis of RCTs (66). There is also some evidence for the potential benefits of JAKi, specifically TOF (67), UPA (68), and filgotinib (69). An investigator-initiated trial (JAKUVEITE) on baricitinib's efficacy for non-infectious uveitis has been registered but has not yet started.^3^ Additionally, brepocitinib is planned to be studied in active non-infectious non-anterior uveitis.^4^ More recently, BKZ has demonstrated a beneficial effect in reducing uveitis recurrence (incidence of 1.8 vs. 15.4 flares per 100 PY) in an analysis of pooled data from ankylosing spondylitis patients included in the BKZ phase 3 BE MOBILE 1 and 2 trials (70). Another systematic literature review and network meta-analysis of available phase 2/3 double-blind RCTs of TNFi monoclonal antibodies (mAb), IL17i (including BKZ), and JAKi in axSpA suggested that all three therapeutic classes might be effective in preventing AAU flares. The incidence rates per 100 person-years were 4.1 for anti-TNF mAb, 5.4 for ETA, 2.8 for anti-IL17, 1.5 for JAKi, and 10.8 for placebo. Considering the surface under the cumulative ranking curve approach to rank AAU risk, the lowest risk was identified for anti-TNF mAbs, followed by JAKi, anti-IL17, and ETA (71). We could not find any literature on the use of a combination of b/tsDMARDs for SpA-associated AAU, likely due to the usually self-limited, less threatening nature of AAU in comparison with intermediate/posterior uveitis. Nevertheless, based on available evidence, it may be reasonable to consider a combination strategy for severe/or recurrent AAU under a single mechanism b/tsDMARD.

## 7 Discussion

Newer treatments and strategies are still required for SpA patients, primarily due to insufficient therapeutic responses, particularly considering the full spectrum of SpA manifestations, primary and secondary failures, and the occurrence of adverse events. Unmet needs in effectively controlling both axial and peripheral disease, such as the ceiling effect of efficacy, ineffective control and prevention of radiographic damage, and low rates of remission, remain to be addressed (6). Additionally, the use of standard pre-defined doses for subcutaneous or oral therapies, according to regulatory approvals, overlooks the fine-tuned management required for personalized medicine. This applies to almost all b/tsDMARDs, with few exceptions, such as IFX. This frequently hinders individual adjustments for those who exhibit more severe MSK symptoms or extra-MSK manifestations within the SpA spectrum, which often require higher dosages of effective therapies. Consequently, this leads to multiple b/tsDMARD switches during follow-up, allowing damage to accumulate and creating a significant financial burden. Faster and more efficacious disease activity suppression that can ideally prevent both MSK and organ damage, ultimately improving patients' quality of life, is therefore needed. In fact, overall disease prevention is a priority for rheumatologists, particularly for PsA patients, acknowledging that early/and effective treatment of Pso can cause the interception of PsA (72).

Current knowledge of physiopathology and experience in treating SpA patients (as well as RA and other inflammatory arthritis) support that disease activity is not dependent on a single cytokine pathway but results from complex interactions among several cellular and molecular players. Therefore, combining the blockade of complementary inflammatory pathways can increase the chances of achieving higher and more sustained remission rates.

The initial trials on dual cytokine blockade, originating from the RA field, led to disappointing results. The combination of ETA and anakinra did not improve efficacy and was associated with an increased risk of severe infections (9). Similarly, the addition of the T cell co-stimulator ABT (2 mg/kg) to ongoing ETA only marginally improved ACR50 responses (even when the dose was increased to 10 mg/kg), and a substantial increase in the rate of serious infections was observed (73). These two trials were followed by a small trial assessing the safety of rituximab added to a TNFi (ETA or ADA) and methotrexate in patients with high disease activity who were refractory. One serious and two grade 3 (non-serious) infections were reported in the combination RTX plus TNFi arm, while none occurred in the TNFi arm. An ACR50 response was observed in 12% (of 33) in the combination arm vs. 6% (of 18) of patients on TNFi monotherapy (74). Another combination trial of RTX added to ADA (67 patients), ETA (65 patients), ABT (26 patients), or IFX (18 patients) in highly active refractory RA patients was also conducted. After the first course of RTX, 31%, 10%, and 5% of patients achieved ACR20, ACR50, and ACR70 responses at week 24, and the safety profile was considered similar to that of adding RTX to csDMARDs (75).

Later, the identification of increased IL17 production by T helper cells in the peripheral blood of RA patients who did not adequately respond to TNFi provided a rationale for evaluating the effect of remtolumab (ABT-122) in RA (19). At 12 weeks, the ACR20 responses were 80% for the ABT-122 120 mg dose arm vs. 68% in the ADA arm, with similar safety profiles. Nonetheless, this difference was considered marginal, and the development of ABT-122 in RA was stopped. More recently, added-on BKZ was evaluated in a short-term (12 weeks) proof-of-concept trial involving RA patients refractory to CZP, after 8 weeks of treatment. This regimen resulted in rates of remission/low disease activity (DAS28CRP < 3.2) in 46% of patients in the combination arm vs. 29% in the CZP arm, with no unexpected safety signals but with an increased rate of treatment-related emergent AEs of 78.8% vs. 59.3% in the combination arm (76). Furthermore, in a systematic literature review and meta-analysis of studies on the combination of two bDMARDs in RA, assessing the rate of SAEs as a primary endpoint, an increase in SAEs in the combination group was reported at 14.9% vs. 6%, and an increase in overall AEs (94.6% vs. 89%) was also noted (77). For these reasons, DTT has not been commonly implemented for the treatment of RA patients in clinical practice.

Despite this evidence from the RA field, the cytokine profile in RA and SpA (and its wide spectrum of manifestations) is distinct, and this has known therapeutic implications. For PsO, more recently developed bDMARDs have been associated with increased efficacy compared to TNFi, but the same does not seem to be true for PsA, where remission is achieved by only a small number of patients (35–42%) (78). For joint manifestations, TNF blockade might not be surpassed by an IL17i or IL23i in monotherapy, as the effect of the former can be predominant, at least in bDMARD-naïve patients. These unmet needs and the increased knowledge of b/tsDMARDs' safety profiles and disease pathophysiology have allowed for some successful approaches to combination strategies in SpA, either based on individual clinical decisions or leading to the development of bispecific biologics or targeted synthetic DMARDs, which is the focus of this review.

In this review, we discuss the most recent evidence about the combination of two approved bDMARDs or of one tsDMARD and one bDMARD, as well as the newly developed bispecific antibodies and targeted synthetic therapies with a dual mode of action. We distinguished bispecific antibodies targeting cytokines with a similar mode of action (MOA) (i.e., IL17A and IL17F) from those targeting cytokines with distinct MOAs (i.e., TNF and IL17). For tsDMARDs, we considered those that block the signaling of two distinct pathways (i.e., JAK and TYK or TEC) as dual-targeted.

Due to the broad spectrum of the subject, the heterogeneity of available studies on DTT in SpA, and limited analytical data (comparing different interventions), a narrative review format was preferred over a systematic review/meta-analysis. This literature search provided a comprehensive overview of all publications reflecting current experience with DTT in SpA. Although it is largely limited to single case reports and case series, it identifies current knowledge gaps and ongoing trials that will likely yield important advances in the near future. However, it is also subject to significant limitations, which reflect the state of the art on this subject. Except for four RCTs (one in axSpA and three in PsA), all available data come from small descriptive studies with no comparator arm, resulting in limited generalizability and a high risk of bias. Indeed, the vast majority of these case reports and case series report very favorable responses to DTT in patients with refractory disease or complex manifestations, likely due to the fact that positive results are more likely to be published (publication bias). Heterogeneous and often short follow-up times also preclude robust conclusions on safety and long-term efficacy. On the other hand, the identified publications show a large heterogeneity of interventions (different types of DTT), indications for DTT (varying degrees of disease activity or extra-MSK manifestations of SpA), efficacy outcomes, and comparators (when available), thus making it impossible to synthesize the evidence in a way that can be immediately applied to clinical decision-making.

From this review, it becomes evident that DTT combinations tend to vary across the spectrum of SpA manifestations. The most common dual anti-cytokine biologic therapy combination identified in SpA with active MSK disease in case reports/and case series was the association of a TNFi and IL12/23i. The presence of concomitant IBD constituted a common indication for DTT, reflecting a more severe/or refractory disease and naturally shifting the prescription preferentially to the IL12/23i class due to the restriction on IL17i utilization. In this subgroup of patients, clinical improvement (despite heterogeneous disease activity measures) was described in a large number of patients exposed. For the PsA/Pso manifestations, TNFi in combination with IL17i/IL23 was more often prescribed, but this population seems to be more frequently refractory even to DTT, with discontinuations due to inefficacy observed in approximately 50% of patients. For IBD as the main manifestation, a large RCT and a prospective study with a meta-analysis of other monotherapy trials as a virtual comparator have recently shown the superiority of DTT combining a TNFi with an IL23i or an anti-integrin over each drug used as monotherapy, with no additional safety concerns. Several case reports and case series also describe promising results for these combinations, as well as the combination of an IL12/23i with an anti-integrin, which might be the safest combination if we extrapolate from the safety profiles of individual bDMARDs.

Due to their often self-limited course and possible association with other more severe SpA manifestations, DTT for specific uveitis indications was not identified in this literature search, with the exception of data from ankylosing spondylitis BKZ trials showing efficacy in the reduction of AAU flares.

It is also worth noting that the majority of patients had previously received one or more bDMARDs, often having failed one of the drugs used in the combination or having been exposed to both, which could have decreased the potential efficacy of DTT.

From a safety perspective, despite initial concerns, discontinuations due to AE were not frequent.

Due to their more recent introduction in the market, DTT with tsDMARDs has been less reported, but some efficacy increment is described for MSK manifestations, namely in axSpA for TOF added to TNFi or in PsA to IL12/23i, IL23i, or IL17i. In IBD, a JAKi in association with an anti-integrin was reported to be almost as effective as a TNFi combined with an anti-integrin, with no serious AE.

For new bispecific antibodies targeting IL17A and IL17F, promising results have been observed, and BKZ is already broadly implemented in clinical practice. On the contrary, the development of bispecific antibodies for TNF and IL17 has been discontinued due to insufficient efficacy. However, it is important to consider that early-phase clinical trials, particularly phase 2, can overestimate efficacy in comparison with later phase 3 trials when interpreting more recent RCT results.

We expect a significant leap in new developments in this field in the coming years. Bispecific antibodies that block two antigens (soluble molecules or cell surface receptors) are being studied across various medical fields. Some bispecific antibodies, however, have had their development halted for different rheumatic diseases, primarily due to inefficacy or safety concerns. This includes lutikizumab (anti-IL1a and IL1b) for osteoarthritis, and COVA322, a bispecific TNF/IL17A antibody fusion protein studied in phase 1/2 in patients with stable chronic moderate-to-severe plaque Pso (stopped for safety reasons). JNJ-61178104 (phase 1), also a bispecific antibody against human tumor necrosis factor and interleukin-17A, was studied in healthy subjects (79, 80). For others, results are not yet available, such as tibulizumab, a BAFF/IL17 bispecific antibody studied in phase 2 in subjects with RA and primary Sjogren's syndrome, which is now being investigated in systemic sclerosis, and MEDI7352, an IgG-like bispecific antibody targeting nerve growth factor and TNF for the treatment of painful osteoarthritis, or obexelimab (targeting CD19 and FcyRIIb) in phase 3 for IgG4-related disease.

Several questions remain to be answered by future studies, including whether combining b/tsDMARDs can reduce secondary failure mechanisms, specifically immunogenicity, and increase drug survival alongside better disease activity control. In addition to long-term studies, RCTs comparing DTT strategies with monotherapy are crucial for improving the quality of evidence in this field. These trials can help clarify the positioning of DTT in the SpA treatment algorithm, especially for difficult-to-treat either or treatment-refractory MSK or extra-MSK manifestations. One additional unresolved issue is the debatable cost-effectiveness of DTT approaches; however, given the direct and indirect costs of persistent high disease activity, it may be reasonable to consider DTT strategies for selected SpA patients.

## Footnotes

^1^ https://www.clinicaltrials.gov/study/NCT05071664?term=guselkumab&intr=Golimumab&rank=2

^2^ https://www.clinicaltrials.gov/search?cond=sonelokimab&checkSpell=

^3^ https://www.clinicaltrials.gov/study/NCT05651880?term=JAKUVEITE&rank=1

^4^ https://www.clinicaltrials.gov/study/NCT05523765?term=brepocitinib&rank=1

## References

[1] Frison E Breban M Costantino F. How to translate genetic findings into clinical applications in spondyloarthritis? Front Immunol. (2024) 15:1–7. 10.3389/fimmu.2024.1301735 38327520 PMC10847566

[2] Robert M Miossec P. Structural cell heterogeneity underlies the differential contribution of IL-17A, IL-17F and IL-23 to joint vs. skin chronic inflammation. Autoimmun Rev. (2024) 23:103529. 10.1016/j.autrev.2024.103529 38492906

[3] Felice C Dal Buono A Gabbiadini R Rattazzi M Armuzzi A. Cytokines in spondyloarthritis and inflammatory bowel diseases: from pathogenesis to therapeutic implications. Int J Mol Sci. (2023) 24:3957. 10.3390/ijms24043957 36835369 PMC9968229

[4] Webers C Ortolan A Sepriano A Falzon L Baraliakos X Landewé RBM. Efficacy and safety of biological DMARDs: A systematic literature review informing the 2022 update of the ASAS-EULAR recommendations for the management of axial spondyloarthritis. Ann Rheum Dis. (2022) 82:130–41. 10.1136/ard-2022-223298 36270657

[5] Poddubnyy D Navarro-Compán V Torgutalp M Arends S Aydin SZ Battista S. The assessment of spondyloarthritis international society (ASAS) consensus-based expert definition of difficult-to-manage, including treatment-refractory, axial spondyloarthritis. Ann Rheum Dis. (2025) 84:8. 10.1016/j.ard.2025.03.008 39955166

[6] Ribeiro AL Proft F. Unraveling the challenges of difficult-to-treat spondyloarthritis: SPARTAN 2024 Annual Meeting Proceedings. Curr Rheumatol Rep. (2025) 27:18. 10.1007/s11926-025-01183-y 39899221 PMC11790757

[7] Ortolan A Webers C Sepriano A Falzon L Baraliakos X Landewé RBM. Efficacy and safety of non-pharmacological and non-biological interventions: A systematic literature review informing the 2022 update of the ASAS/EULAR recommendations for the management of axial spondyloarthritis. Ann Rheum Dis. (2022) 82:142–52. 10.1136/ard-2022-223297 36261247

[8] Kerschbaumer A Smolen JS Ferreira RJO Bertheussen H Baraliakos X Aletaha D. Efficacy and safety of pharmacological treatment of psoriatic arthritis: a systematic literature research informing the 2023 update of the EULAR recommendations for the management of psoriatic arthritis. Ann Rheum Dis. (2024) 83:760–74. 10.1136/ard-2024-225534 38503473 PMC11103324

[9] Genovese MC Cohen S Moreland L Lium D Robbins S Newmark R. Combination therapy with etanercept and anakinra in the treatment of patients with rheumatoid arthritis who have been treated unsuccessfully with methotrexate. Arthritis Rheum. (2004) 50:1412–9. 10.1002/art.20221 15146410

[10] Valero-Martínez C Urgelles JF Sallés M Joven-Ibáñez BE de Juanes A Ramírez J. Dual targeted therapy in patients with psoriatic arthritis and spondyloarthritis: a real-world multicenter experience from Spain. Front Immunol. (2023) 14:1–12. 10.3389/fimmu.2023.1283251 37936691 PMC10627177

[11] Adams R Maroof A Baker T Lawson ADG Oliver R Paveley R. Bimekizumab, a novel humanized IgG1 antibody that neutralizes both IL-17A and IL-17F. Front Immunol. (2020) 11:1894. 10.3389/fimmu.2020.01894 32973785 PMC7473305

[12] Baraliakos X Deodhar A Van Der Heijde D Magrey M Maksymowych WP Tomita T. Bimekizumab treatment in patients with active axial spondyloarthritis: 52-week efficacy and safety from the randomised parallel phase 3 BE MOBILE 1 and BE MOBILE 2 studies. Ann Rheum Dis. (2023) 11:199–213. 10.1136/ard-2023-224803 37793792

[13] Coates LC Landewé R McInnes IB Mease PJ Ritchlin CT Tanaka Y. Bimekizumab treatment in patients with active psoriatic arthritis and prior inadequate response to tumour necrosis factor inhibitors: 52-week safety and efficacy from the phase III BE COMPLETE study and its open-label extension BE VITAL. RMD Open. (2024) 10:e003855. 10.1136/rmdopen-2023-003855 38388171 PMC10884206

[14] Deodhar A Machado PM Mørup M Taieb V Willems D Orme M. Comparative efficacy and safety of bimekizumab in axial spondyloarthritis: a systematic literature review and network meta-Analysis. Rheumatol. (2024) 63:1195–205. 10.1093/rheumatology/kead598 37947318 PMC11065447

[15] Maksymowych WP Thom H Mørup MF Taieb V Willems D Lyris N. Matching-adjusted indirect comparison of the 52-week efficacy of bimekizumab vs. secukinumab and ixekizumab for the treatment of radiographic axial spondyloarthritis. Rheumatol Ther. (2024) 11:1023–41. 10.1007/s40744-024-00684-z 38916823 PMC11265043

[16] Mease PJ Genovese MC Weinblatt ME Peloso PM Chen K Othman AA. Phase II study of ABT-122, a tumor necrosis factor– and interleukin-17A–targeted dual variable domain immunoglobulin, in patients with psoriatic arthritis with an inadequate response to methotrexate. Arthritis Rheumatol. (2018) 70:1778–89. 10.1002/art.40579 29855175 PMC6221045

[17] Cuchacovich R Garcia-Valladares I Espinoza LR. Combination biologic treatment of refractory psoriasis. J Rheumatol. (2012) 39:187–93. 10.3899/jrheum.110295 22210681

[18] Simon D Fagni F Schett G. Sequential interleukin-17/interleukin-23 inhibition in treatment-refractory psoriatic arthritis. Ann Rheum Dis. (2022) 81:1334–6. 10.1136/annrheumdis-2022-222415 35512847

[19] Genovese MC Weinblatt ME Aelion JA Mansikka HT Peloso PM Chen K. ABT-122, a bispecific dual variable domain immunoglobulin targeting tumor necrosis factor and interleukin-17A, in patients with rheumatoid arthritis with an inadequate response to methotrexate: a randomized, double-blind study. Arthritis Rheumatol. (2018) 70:1710–20. 10.1002/art.40580 29855172 PMC6704363

[20] Genovese MC Weinblatt ME Mease PJ Aelion JA Peloso PM Chen K. Dual inhibition of tumour necrosis factor and interleukin-17A with ABT-122: Open-label long-term extension studies in rheumatoid arthritis or psoriatic arthritis. Rheumatol. (2018) 57:1972–81. 10.1093/rheumatology/key173 30032191

[21] Gordon KB Foley P Krueger JG Pinter A Reich K Vender R. Bimekizumab efficacy and safety in moderate to severe plaque psoriasis (BE READY): a multicentre, double-blind, placebo-controlled, randomised withdrawal phase 3 trial. Lancet. (2021) 397:475–86. 10.1016/S0140-6736(21)00126-4 33549192

[22] Strober B Paul C Blauvelt A Thaçi D Puig L Lebwohl M. Bimekizumab efficacy and safety in patients with moderate to severe plaque psoriasis: Two-year interim results from the open-label extension of the randomized BE RADIANT phase 3b trial. J Am Acad Dermatol. (2023) 89:486–95. 10.1016/j.jaad.2023.04.063 37182701

[23] Strober B Tada Y Mrowietz U Lebwohl M Foley P Langley RG. Bimekizumab maintenance of response through 3 years in patients with moderate-to-severe plaque psoriasis: results from the BE BRIGHT open-label extension trial. Br J Dermatol. (2023) 188:749–59. 10.1093/bjd/ljad035 36967713

[24] Reich K Papp KA Blauvelt A Langley RG Armstrong A Warren RB. Bimekizumab vs. ustekinumab for the treatment of moderate to severe plaque psoriasis (BE VIVID): efficacy and safety from a 52-week, multicentre, double-blind, active comparator and placebo controlled phase 3 trial. Lancet. (2021) 397:487–98. 10.1016/S0140-6736(21)00125-2 33549193

[25] Rompoti N Stefanaki I Panagakis P Vavouli C Politou M Papoutsaki M. Bimekizumab in psoriasis: a monocentric study evaluating short- and mid-term effectiveness and safety profile in a real-world setting. Arch Dermatol Res. (2024) 316:133. 10.1007/s00403-024-02868-7 38662223

[26] Gargiulo L Narcisi A Ibba L Balato A Bianchi L Brianti P. Effectiveness and safety of bimekizumab for the treatment of plaque psoriasis: a real-life multicenter study—IL PSO (Italian landscape psoriasis). Front Med. (2023) 10:8–12. 10.3389/fmed.2023.1243843 37614958 PMC10442506

[27] Sayed C Shi V Hsiao J Kokolakis G Kirby B Piguet V. Bimekizumab efficacy by prior biologic treatment in patients with moderate to severe hidradenitis suppurativa: 48-week pooled data from the randomized, double-blind, placebo-controlled, multicenter BE HEARD I and II phase 3 trials. Ski J Cutan Med. (2024) 8:s349. 10.25251/skin.8.supp.349

[28] Ritchlin CT Kavanaugh A Merola JF Schett G Scher JU Warren RB. Bimekizumab in patients with active psoriatic arthritis: results from a 48-week, randomised, double-blind, placebo-controlled, dose-ranging phase 2b trial. Lancet. (2020) 395:427–40. 10.1016/S0140-6736(19)33161-7 32035552

[29] Ritchlin CT Coates LC McInnes IB Mease PJ Merola JF Tanaka Y. Bimekizumab treatment in biologic DMARD-naïve patients with active psoriatic arthritis: 52-week efficacy and safety results from the phase III, randomised, placebo-controlled, active reference BE OPTIMAL study. Ann Rheum Dis. (2023) 82:1404–14. 10.1136/ard-2023-224431 37696588 PMC10579478

[30] Warren RB McInnes IB Nash P Grouin J-M Lyris N Willems D. Comparative effectiveness of bimekizumab and guselkumab in patients with psoriatic arthritis at 52 weeks assessed using a matching-adjusted indirect comparison. Rheumatol Ther Junho de. (2024) 11:829–39. 10.1007/s40744-024-00659-0 38488975 PMC11111623

[31] Mease PJ Gladman DD Merola JF Nash P Grieve S Laliman-Khara V. Comparative efficacy and safety of bimekizumab in psoriatic arthritis: a systematic literature review and network meta-analysis. Rheumatology. (2024) 63:1779–89. 10.1093/rheumatology/kead705 38218744 PMC11215990

[32] Svecova D Lubell MW Casset-Semanaz F Mackenzie H Grenningloh R Krueger JG. randomized, double-blind, placebo-controlled phase 1 study of multiple ascending doses of subcutaneous M1095, an anti–interleukin 17A/F nanobody, in moderate-to-severe psoriasis. J Am Acad Dermatol. (2019) 81:196–203. 10.1016/j.jaad.2019.03.056 30926369

[33] Papp KA Weinberg MA Morris A Reich K. IL17A/F nanobody sonelokimab in patients with plaque psoriasis: a multicentre, randomised, placebo-controlled, phase 2b study. Lancet. (2021) 397:1564–75. 10.1016/S0140-6736(21)00440-2 33894834

[34] Gyldenløve M Alinaghi F Zachariae C Skov L Egeberg A. Combination therapy with apremilast and biologics for psoriasis: a systematic review. Am J Clin Dermatol. (2022) 23:605–13. 10.1007/s40257-022-00703-1 35737251

[35] Takamura S Sugai S Taguchi R Teraki Y. Combination therapy of apremilast and biologics in patients with psoriasis showing biologic fatigue. J Dermatol. (2020) 47:290–4. 10.1111/1346-8138.15193 31867729

[36] Abignano G Fadl N Merashli M Wenham C Freeston J McGonagle D. Apremilast for the treatment of active psoriatic arthritis: a single-centre real-life experience. Rheumatol. (2018) 57:578–80. 10.1093/rheumatology/kex454 29272544 PMC5850831

[37] Metyas S Tomassian C Messiah R Gettas T Chen C Quismorio A. Combination therapy of apremilast and biologic agent as a safe option of psoriatic arthritis and psoriasis. Curr Rheumatol Rev. (2019) 15:234–7. 10.2174/1573397115666181130094455 30499418

[38] Barroso NS Miller EZ Furst DE. A case series on patients on tofacitinib in combination with a biologic. J Clin Rheumatol. (2018) 24:349–51. 10.1097/RHU.0000000000000663 29280829

[39] Shurey M Yip A Ziouzina O Chan J Dutz JP. Combination therapy with tofacitinib and IL-12/23, IL-23, or IL-17A inhibition for the treatment of refractory psoriatic arthritis: a case series. J Clin Rheumatol. (2022) 28:e626–8. 10.1097/RHU.0000000000001767 34176886

[40] Guénin S Andrews E Lebwohl MG. Safety and efficacy of dual tyrosine kinase 2 inhibitor and monoclonal antibody therapy for psoriasis and psoriatic arthritis. Br J Dermatol. (2024) 190:451–3. 10.1093/bjd/ljad473 38011328

[41] Mease P Helliwell P Silwinska-Stanczyk P Miakisz M Ostor A Peeva E. Efficacy and safety of the TYK2/JAK1 inhibitor brepocitinib for active psoriatic arthritis: a phase IIb randomized controlled trial. Arthritis Rheumatol. (2023) 75:1370–80. 10.1002/art.42519 37194394

[42] Sands BE Kozarek R Spainhour J Barish CF Becker S Goldberg L. Safety and tolerability of concurrent natalizumab treatment for patients with Crohn's disease not in remission while receiving infliximab. Inflamm Bowel Dis. (2007) 13:2–11. 10.1002/ibd.20014 17206633

[43] Feagan BG Sands BE Sandborn WJ Germinaro M Vetter M Shao J. Guselkumab plus golimumab combination therapy vs. guselkumab or golimumab monotherapy in patients with ulcerative colitis (VEGA): a randomised, double-blind, controlled, phase 2, proof-of-concept trial. Lancet Gastroenterol Hepatol. (2023) 8:307–20. 36738762 10.1016/S2468-1253(22)00427-7

[44] Yang E Panaccione N Whitmire N Dulai PS Vande Casteele N Singh S. Efficacy and safety of simultaneous treatment with two biologic medications in refractory Crohn's disease. Aliment Pharmacol Ther. (2020) 51:1031–8. 10.1111/apt.15719 32329532 PMC8032452

[45] Eronen H Kolehmainen S Koffert J Koskinen I Oksanen P Jussila A. Combining biological therapies in patients with inflammatory bowel disease: a Finnish multi-centre study. Scand J Gastroenterol. (2022) 57:936–41. 10.1080/00365521.2022.2045350 35238727

[46] Yzet C Dupas J-L Fumery M. Ustekinumab and Anti-TNF combination therapy in patients with inflammatory bowel disease. Off J Am Coll Gastroenterol. (2016) 111:748. 10.1038/ajg.2016.66 27151127

[47] Huff-Hardy K Bedair M Vazquez R Burstein E. Efficacy of combination vedolizumab and ustekinumab for refractory Crohn's disease. Inflamm Bowel Dis. (2017) 23:E49. 10.1097/MIB.0000000000001232 28858074

[48] O'Toole A Lucci M Korzenik J. Inflammatory bowel disease provoked by etanercept: report of 443 possible cases combined from an IBD referral center and the FDA. Dig Dis Sci. (2016) 61:1772–4. 10.1007/s10620-015-4007-z 26728477

[49] Onac IA Clarke BD Tacu C Lloyd M Hajela V Batty T. Secukinumab as a potential trigger of inflammatory bowel disease in ankylosing spondylitis or psoriatic arthritis patients. Rheumatology. (2021) 60:5233–8. 10.1093/rheumatology/keab193 33677579

[50] Huseynzada S Yüce Inel T Hajiyev F Köken Avşar A Balci A Akpinar H. The effect of vedolizumab on spondyloarthritis symptoms in a cohort of inflammatory bowel disease patients. Eur J Rheumatol. (2023) 10:50–6. 10.5152/eurjrheum.2023.22049 37171478 PMC10542484

[51] Bethge J Meffert S Ellrichmann M Conrad C Nikolaus S Schreiber S. Combination therapy with vedolizumab and etanercept in a patient with pouchitis and spondylarthritis. BMJ Open Gastroenterol. (2017) 4:e000127. 10.1136/bmjgast-2016-000127 28243458 PMC5306500

[52] Richard N Hazel EM Haroon N Inman RD. Simultaneous inhibition of α4/β7 integrin and tumour necrosis factor-α in concomitant spondyloarthritis and inflammatory bowel disease. Ann Rheum Dis. (2018) 77:e86–e86. 10.1136/annrheumdis-2017-212819 29288208

[53] Bass J Goyal A. P012 successful use of combination biologic therapy in medically refractory pediatric Crohn's disease and sacroiliitis. Off J Am Coll Gastroenterol. (2019) 114:S3. 10.14309/01.ajg.0000613016.81984.79

[54] Elmoursi A Barrett TA Perry C. Double biologic therapy for refractory stricturing crohn's disease: a successful case of deep remission with ustekinumab and vedolizumab. Inflamm Bowel Dis. (2020) 26:e62–3. 10.1093/ibd/izaa092 32386054 PMC7301404

[55] Caron B Netter P Danese S Peyrin-Biroulet L. Bispecific antibodies for the treatment in inflammatory bowel disease: an avenue worth exploring? Expert Opin Biol Ther. (2022) 22:951–3. 10.1080/14712598.2022.1985999 34612123

[56] Yin Q Pi X Jiang Y Ren G Liu Z Liu H. An immuno-blocking agent targeting IL-1β and IL-17A reduces the lesion of DSS-induced ulcerative colitis in mice. Inflammation. (2021) 44:1724–36. 10.1007/s10753-021-01449-4 33877484

[57] Glassner K Oglat A Duran A Koduru P Perry C Wilhite A. The use of combination biological or small molecule therapy in inflammatory bowel disease: a retrospective cohort study. J Dig Dis 5. (2020) 21:264–71. 10.1111/1751-2980.12867 32324969

[58] Goessens L Colombel J-F Outtier A Ferrante M Sabino J Judge C. Safety and efficacy of combining biologics or small molecules for inflammatory bowel disease or immune-mediated inflammatory diseases: a European retrospective observational study. United Eur Gastroenterol J. (2021) 9:1136–47. 10.1002/ueg2.12170 34694746 PMC8672088

[59] Miyatani Y Choi D Choi NK Rubin DT. Dual-targeted therapy with upadacitinib and ustekinumab in medically complex Crohn's disease. Dig Dis Sci. (2024) 69:355–9. 10.1007/s10620-023-08182-y 38112840

[60] Le Berre C Loeuille D Peyrin-Biroulet L. Combination therapy with vedolizumab and tofacitinib in a patient with ulcerative colitis and spondyloarthropathy. Clin Gastroenterol Hepatol. (2019) 17:794–6. 10.1016/j.cgh.2018.08.017 30114486

[61] Rubin DT Cleveland NK. Use of tofacitinib for the treatment of arthritis associated with vedolizumab in ulcerative colitis: 1987. Off J Am Coll Gastroenterol. (2017) 112:S1098. 10.14309/00000434-201710001-01988

[62] Sandborn WJ Danese S Leszczyszyn J Romatowski J Altintas E Peeva E. Oral ritlecitinib and brepocitinib for moderate-to-severe ulcerative colitis: results from a randomized, phase 2b study. Clin Gastroenterol Hepatol. (2023) 21:2616–28.e7. 10.1016/j.cgh.2022.12.029 36623678

[63] Vermeire S Allegretti JR Kim HJ et al. OP09 Oral ritlecitinib and brepocitinib in patients with moderate to severe active Crohn's Disease: data from the PIZZICATO umbrella study. J Crohn's Colitis. (2024) 18:9. 10.1093/ecco-jcc/jjad212.0009

[64] Calvo-Río V Blanco R Santos-Gómez M Rubio-Romero E Cordero-Coma M Gallego-Flores A. Golimumab in refractory uveitis related to spondyloarthritis. Multicenter study of 15 patients. Semin Arthritis Rheum. (2016) 46:95–101. 10.1016/j.semarthrit.2016.03.002 27060872

[65] Van Der Horst-Bruinsma I Van Bentum R Verbraak FD Rath T Rosenbaum JT Misterska-Skora M. The impact of certolizumab pegol treatment on the incidence of anterior uveitis flares in patients with axial spondyloarthritis: 48-week interim results from C-VIEW. RMD Open. (2020) 6:1–9. 10.1136/rmdopen-2019-001161 32371433 PMC7299504

[66] Roche D Badard M Boyer L Lafforgue P Pham T. Incidence of anterior uveitis in patients with axial spondyloarthritis treated with anti-TNF or anti-IL17A : a systematic review, a pairwise and network meta-analysis of randomized controlled trials. Arthritis Res Ther. (2021) 23:192. 10.1186/s13075-021-02549-0 34271991 PMC8283999

[67] Gupta N MD. AB1375 tofacitinib as alternative to tnf blockers in refractory anterior uveitis - an observational study. Ann Rheum Dis. (2024) 83:2040. 10.1136/annrheumdis-2024-eular.304

[68] Van Der Heijde D Baraliakos X Sieper J Deodhar A Inman RD Kameda H. Efficacy and safety of upadacitinib for active ankylosing spondylitis refractory to biological therapy: a double-blind, randomised, placebo-controlled phase 3 trial. Ann Rheum Dis. (2022) 81:1515–23. 10.1136/ard-2022-222608 35788492 PMC9606523

[69] Srivastava SK Watkins TR Nguyen QD Sharma S Scales DK Dacey MS. Filgotinib in active noninfectious uveitis: the HUMBOLDT randomized clinical trial. JAMA Ophthalmol. (2024) 142:789–97. 10.1001/jamaophthalmol.2024.2439 39023880 PMC11258638

[70] Brown MA Rudwaleit M van Gaalen FA Haroon N Gensler LS Fleurinck C. Low uveitis rates in patients with axial spondyloarthritis treated with bimekizumab: pooled results from phase 2b/3 trials. Ann Rheum Dis. (2024) 83:1722–30. 10.1136/ard-2024-225933 38977276 PMC12056585

[71] Bechman K Yang Z Adas M Nagra DS Uguzlar A Russell MD. Incidence of uveitis in patients with axial spondylarthritis treated with biologics or targeted synthetics: a systematic review and network meta-analysis. Arthritis Rheumatol. (2024) 76:704–14. 10.1002/art.42788 38116697

[72] López-Medina C McGonagle D Gossec L. Subclinical psoriatic arthritis and disease interception—where are we in 2024? Rheumatology. (2024) 3:56–64. 10.1093/rheumatology/keae399 39150442 PMC11701312

[73] Weinblatt M Schiff M Goldman A Kremer J Luggen M Li T. Selective costimulation modulation using abatacept in patients with active rheumatoid arthritis while receiving etanercept: a randomised clinical trial. Ann Rheum Dis. (2007) 66:228–34. 10.1136/ard.2006.055111 16935912 PMC1798511

[74] Greenwald MW Shergy WJ Kaine JL Sweetser MT Gilder K Linnik MD. Evaluation of the safety of rituximab in combination with a tumor necrosis factor inhibitor and methotrexate in patients with active rheumatoid arthritis: results from a randomized controlled trial. Arthritis Rheum. (2011) 63:622–32. 10.1002/art.30194 21360491

[75] Rigby WFC Mease PJ Olech E Ashby M Tole S. Safety of rituximab in combination with other biologic disease-modifying antirheumatic drugs in rheumatoid arthritis: an open-label study. J Rheumatol. (2013) 40:599–604. 10.3899/jrheum.120924 23547218

[76] Glatt S Taylor PC Mcinnes IB Schett G Landewé R Baeten D. Efficacy and safety of bimekizumab as add-on therapy for rheumatoid arthritis in patients with inadequate response to certolizumab pegol: a proof-of-concept study. Ann Rheum Dis. (2019) 78:1033–40. 10.1136/annrheumdis-2018-214943 31177099 PMC6691864

[77] Boleto G Kanagaratnam L Dramé M Salmon JH. Safety of combination therapy with two bDMARDs in patients with rheumatoid arthritis: a systematic review and meta-analysis. Semin Arthritis Rheum. (2019) 49:35–42. 10.1016/j.semarthrit.2018.12.003 30638975

[78] Zardin-Moraes M Da Silva ALFA Saldanha C Kohem CL Coates LC Henrique LR. Prevalence of psoriatic arthritis patients achieving minimal disease activity in real-world studies and randomized clinical trials: Systematic review with metaanalysis. J Rheumatol. (2020) 47:839–46. 10.3899/jrheum.190677 31575702

[79] Zhao Q. Bispecific antibodies for autoimmune and inflammatory diseases : clinical progress to date. BioDrugs. (2020) 34:111–9. 10.1007/s40259-019-00400-2 31916225

[80] Liu Z Song L Yang J Liu H Zhang Y Pi X. Discovery and preclinical evaluation of KYS202004A, a novel bispecific fusion protein targeting TNF-α and IL-17A, in autoimmune disease models. Int Immunopharmacol. (2024) 136:112383. 10.1016/j.intimp.2024.112383 38843642

[81] Lowes MA Turton JA Krueger JG Barnetson RSC. Psoriasis vulgaris flare during efalizumab therapy does not preclude future use: a case series. BMC Dermatol. (2005) 5:1–6. 10.1186/1471-5945-5-9 16109173 PMC1208875

[82] Hamilton TK. Treatment of psoriatic arthritis and recalcitrant skin disease with combination therapy. J Drugs Dermatol. (2008) 7:1089−93. 19110745

[83] Adişen E Karaca F Gürer MA. When there is no single best biological agent: Psoriasis and psoriatic arthritis in the same patient responding to two different biological agents. Clin Exp Dermatol. (2008) 33:164–6. 10.1111/j.1365-2230.2007.02673.x 18257837

[84] Kitamura G Mehr N Anderson N Sirichotiratana M A. case of tuberculosis in a patient on Efalizumab and Etanercept for treatment of refractory palmopustular psoriasis and psoriatic arthritis. Dermatol Online J. (2009) 15:11. 10.5070/D31HD646HW 19336028

[85] Heinecke GM Luber AJ Levitt JO Lebwohl MG. Combination use of ustekinumab with other systemic therapies: a retrospective study in a tertiary referral center. J Drugs Dermatol. (2013) 12:1098–102. 24085044

[86] Babalola O Lakdawala N Strober BE. Combined biologic therapy for the treatment of psoriasis and psoriatic arthritis: a case report. JAAD Case Reports. (2015) 1:3–4. 10.1016/j.jdcr.2014.09.002 27075123 PMC4802563

[87] Gniadecki R Bang B Sand C. Combination of antitumour necrosis factor-α and anti-interleukin-12/23 antibodies in refractory psoriasis and psoriatic arthritis: a long-term case-series observational study. Br J Dermatol. (2016) 174:1145–6. 10.1111/bjd.14270 26522308

[88] Torre KM Payette MJ. Combination biologic therapy for the treatment of severe palmoplantar pustulosis. JAAD Case Reports. (2017) 3:240–2. 10.1016/j.jdcr.2017.03.002 28540359 PMC5432680

[89] De Marco G McGonagle D Mathieson HR Merashli M Magee C FitzGerald O. Combined inhibition of tumour necrosis factor-alpha and interleukin-12/23 for long-standing, refractory psoriatic disease: a differential role for cytokine pathways? Rheumatol. (2018) 57:2053–5. 10.1093/rheumatology/key199 29982709

[90] Rathod D Weinberg JM Yamauchi PS Kircik LH Wollina U Lotti T. Successful treatment of refractory plaque-type psoriasis and psoriatic arthritis with guselkumab and adalimumab combination therapy: a case report. J Drugs Dermatol. (2019) 18:394–6. 31017383

[91] Thibodeaux Q Ly K Reddy V Smith MP Liao W. Dual biologic therapy for recalcitrant psoriasis and psoriatic arthritis. JAAD Case Reports. (2019) 5:928–30. 10.1016/j.jdcr.2019.08.015 31649982 PMC6804560

[92] Hanna S Youssef P Lowe P. Novel combination biologic therapy for recalcitrant psoriasis and psoriatic arthritis in a medically complex patient. Australas J Dermatol. (2022) 63:e63–6. 10.1111/ajd.13752 34813085

[93] Hirten R Longman RS Bosworth BP Steinlauf A Scherl E. Vedolizumab and infliximab combination therapy in the treatment of Crohn's disease. Off J Am Coll Gastroenterol. (2015) 110:1737. 10.1038/ajg.2015.355 26673509

[94] Liu EY Loomes DE. Ustekinumab and vedolizumab dual biologic therapy in the treatment of Crohn's disease. Case Rep Med. (2017) 2017:5264216. 10.1155/2017/5264216 29250117 PMC5698793

[95] Fischer S Rath T Geppert C-I Manger B Schett G Neurath MF. Long-term combination therapy with anti-TNF plus vedolizumab induces and maintains remission in therapy-refractory Ulcerative colitis. Off J Am Coll Gastroenterol. (2017) 112:1621. 10.1038/ajg.2017.242 28978957

[96] Buer LCT Høivik ML Warren DJ Medhus AW Moum BA. Combining anti-TNF-α and vedolizumab in the treatment of inflammatory bowel disease: a case series. Inflamm Bowel Dis. (2018) 24:997–1004. 10.1093/ibd/izx110 29668901

[97] Mao EJ Lewin S Terdiman JP Beck K. Safety of dual biological therapy in Crohn's disease: a case series of vedolizumab in combination with other biologics. BMJ open Gastroenterol. (2018) 5:e000243. 10.1136/bmjgast-2018-000243 30538822 PMC6254738

[98] Roblin X Paul S Ben-Horin S. Co-treatment with golimumab and vedolizumab to treat severe UC and associated spondyloarthropathy. J Crohns Colitis. (2018) 12:379–80. 10.1093/ecco-jcc/jjx142 29088342

[99] Cline A Pichardo RO. Successful treatment of hidradenitis suppurativa in the setting of Crohn disease with combination adalimumab and ustekinumab. Dermatol Online J. (2019) 25:45519. 10.5070/D3259045519 31738849

[100] Privitera G Onali S Pugliese D Renna S Savarino E Viola A. Dual targeted therapy: a possible option for the management of refractory inflammatory bowel disease. J Crohns Colitis. (2020) 2020:jjaa149. 10.1093/ecco-jcc/jjaa149 32674156

[101] Biscaglia G Piazzolla M Cocomazzi F Melchionda G De Cata A Bossa F. Landmarks for dual biological therapy in inflammatory bowel disease: lesson from two case reports of vedolizumab in combination with ustekinumab. Eur J Gastroenterol Hepatol. (2020) 32:1579–82. 10.1097/MEG.0000000000001919 32947419

[102] Fumery M Yzet C Brazier F. Letter: combination of biologics in inflammatory bowel diseases. Aliment Pharmacol Ther. (2020) 52:566–7. 10.1111/apt.15891 32656825

[103] Kwapisz L Raffals LE Bruining DH Pardi DS Tremaine WJ Kane S V. Combination biologic therapy in inflammatory bowel disease: experience from a tertiary care center. Clin Gastroenterol Hepatol Off Clin Pract J Am Gastroenterol Assoc. (2021) 19:616–7. 10.1016/j.cgh.2020.02.017 32068149

[104] Colombel J-F Ungaro RC Sands BE Siegel CA Wolf DC Valentine JF. Vedolizumab, adalimumab, and methotrexate combination therapy in Crohn's disease (EXPLORER). Clin Gastroenterol Hepatol Off Clin Pract J Am Gastroenterol Assoc. (2024). 22:1487–96. 10.1016/j.cgh.2023.09.010 37743037

